# Applications of the wheat germ cell-free protein synthesis system in plant biochemical studies

**DOI:** 10.5511/plantbiotechnology.24.0501a

**Published:** 2024-12-25

**Authors:** Keiichirou Nemoto

**Affiliations:** 1 Iwate Biotechnology Research Center, 22-174-4 Narita, Kitakami, Iwate 024-0003, Japan

**Keywords:** chemical screening, high-throughput screening, protein array, wheat germ cell-free system

## Abstract

The development of cell-free protein synthesis technology has made it possible to easily and quickly synthesize recombinant proteins. Among cell-free protein synthesis systems, wheat germ cell-free protein synthesis using eukaryotic ribosomes is an efficient approach to synthesize proteins with diverse and complex structures and functions. However, to date, cell-free protein synthesis systems, including wheat germ cell-free systems, have not been widely used in plant research, and little is known about their applications. Here, I first introduce a basic overview of the cell-free protein synthesis system of wheat germ. Next, I will focus on our previous research examples on plants and present the applications in which the wheat germ cell-free system is used. We provide protein expression and protein function screening methods at the semi-genomic level and also introduce new approaches to enhance study of chemical biology by adapting the cell-free system of wheat germ. With this review, I would like to highlight the potential of the wheat germ cell-free system and position it as a widely used tool for the previously difficult task of recombinant protein preparation and functional analysis.

## Introduction

Numerous studies, mainly focused on model plants, have revealed the functions of important genes involved in the control of various physiological and ecological processes in plants. Using this information, synthetic biology approaches led to the improvement of crop traits, including production of valuable substances and disease resistance (Garland and Curry 2022; Liu et al. 2023; Zhang et al. 2014). Furthermore, there is increasing interest in using plants as platforms for producing recombinant proteins and small molecules for industrial and medical purposes (Coates et al. 2022; Guo et al. 2022; Liu et al. 2023; Malaquias et al. 2021). To achieve many of these applications, it is essential to rapidly characterize the functions of target proteins. However, researchers often spend considerable time and effort to obtain recombinant proteins that retain functionality for in vitro assays. In many cases, living cell systems such as *Escherichia coli* (*E. coli*) are used for synthesizing recombinant proteins. However, it is difficult to synthesize proteins that affect physiological responses of host cells, such as signal transduction factors, membrane proteins, transcription factors, and toxins. This problem represents a significant bottleneck in the analysis of protein function.

In 1954, a method for in vitro protein synthesis using rat liver extracts was reported (Zamecnik and Keller 1954). Subsequently, a comprehensive systematic optimization of cell-free translation systems was carried out using extracts from *E. coli* and *wheat* germ, establishing them as a practical protein synthesis technology (Kigawa et al. 2004; Liu et al. 2005; Madin et al. 2000; Schwarz et al. 2007; Takai et al. 2010). In addition, in recent years, cell-free translation systems based on HeLa (Mikami et al. 2008), Chinese hamster ovary (Brödel et al. 2015), *Saccharomyces cerevisiae* (Gan and Jewett 2014), *Pichia pastoris* (Spice et al. 2020), *tobacco* BY-2 cells (Buntru et al. 2015), and rice (Suzuki et al. 2020) are also being developed. Cell-free systems have been used primarily for protein functional analysis, proteomics, and structural biology, but in recent years, they have emerged as powerful tools in a variety of research areas such as synthetic biology and bioengineering (Fogeron et al. 2021; Harbers 2014; Jiang et al. 2018; Yue et al. 2023). Therefore, this remarkable technology can improve life science research.

Despite this versatility, cell-free systems have not yet been widely applied in plant science, partly due to the complexity of producing cell-free systems. However, in recent years, cell-free systems based on *wheat* germ and *E. coli* have become commercially available, and the possible uses for them are expanding. Therefore, in this paper, we will present research examples of the application of the wheat germ cell-free system in plant science research, focusing on our previous studies.

## Protein expression with wheat germ cell-free system

**Figure 1:**
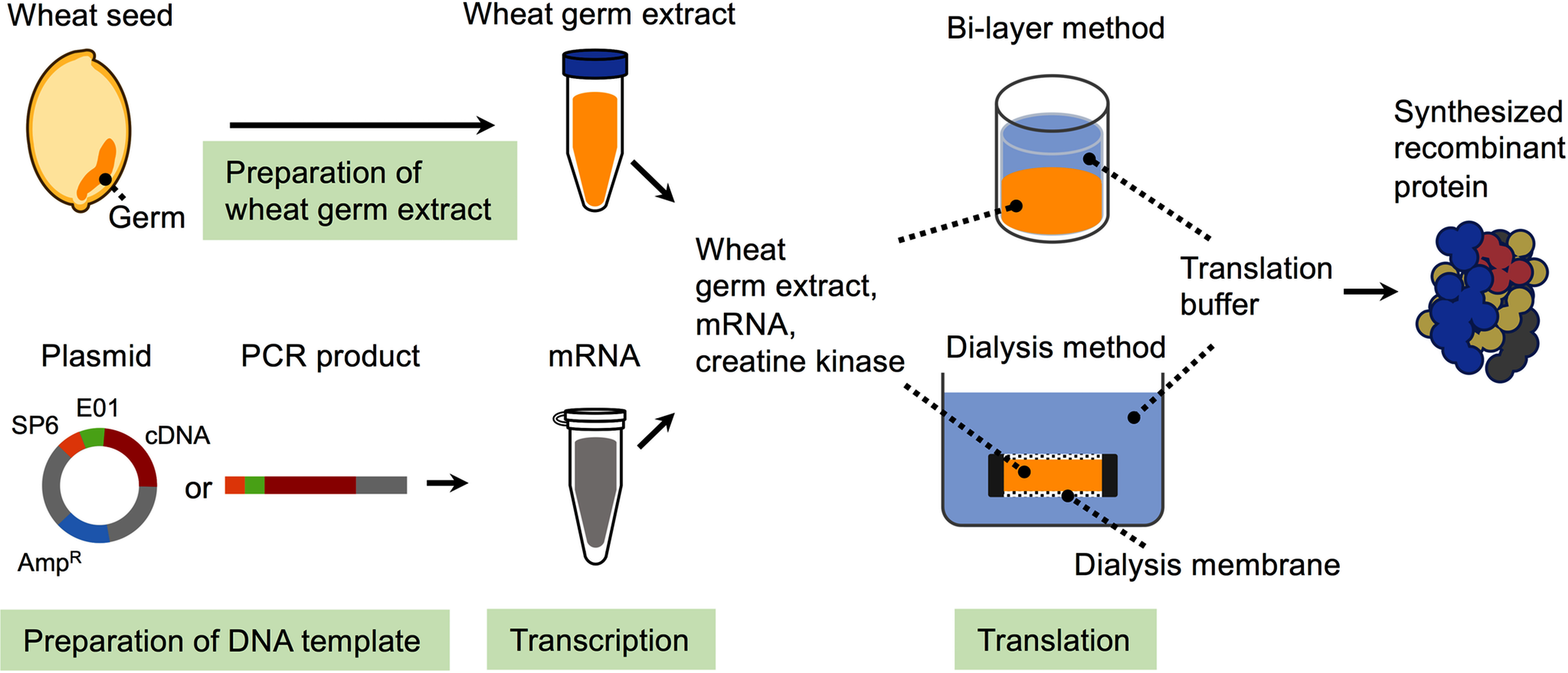
Figure 1. Overview of wheat germ cell-free protein synthesis system. Expression templates for proteins expressed in the wheat germ cell-free protein synthesis system are prepared by expression plasmids or direct PCR amplification. The expression template contains the SP6 RNA polymerase promoter (shown in orange) and the translation enhancer region E01 (shown in green) upstream of the cDNA open reading frame (shown in red). Gray and blue color regions indicate plasmid-derived regions and selection markers on the plasmid, respectively. First, mRNA is synthesized through an in vitro transcription reaction. Subsequently, the synthesized mRNA is mixed with wheat germ extract and creatine kinase, and translation reactions are carried out using a format such as a bi-layer method or a dialysis method. Protein synthesis reactions rapidly reduce adenosine triphosphate (ATP), but creatine kinase and phosphocreatine provide the ATP regeneration necessary to support protein expression. Translation buffer contains components such as amino acids and ATP that are required for the translation reaction.

Wheat germ extract has been used in cell-free translation systems for many years, but they have been unstable and inefficient (Roberts and Paterson 1973). Endo and colleagues investigated the destabilizing factors of the cell-free protein synthesis system of wheat germ. They found that the cause was translation inhibitory factors such as ribosome-inactivating proteins, nucleases, and proteases, which contaminate the embryonic extract from the endosperm during the extraction process and led to destabilization of the translation reaction. Therefore, they developed a method to remove translation inhibitory factors and established a practical technology for the stable synthesis of proteins (Madin et al. 2000). Furthermore, expression vectors optimal for wheat germ cell-free systems and techniques for direct construction of matrices using PCR have been developed, the use of which allows maximizing the performance of the wheat germ cell-free system (Sawasaki et al. 2002b). On the other hand, protein synthesis has been difficult to achieve using conventional batch methods over a long period of time due to the depletion of amino acids and energy sources and the accumulation of byproducts. To solve this problem, various reaction formats, such as bi-layers and dialysis methods, were developed, resulting in drastically improved yields (Figure 1) (Sawasaki et al. 2002a; Spirin et al. 1988). The yield varies depending on the protein to be synthesized and the synthesis method, but in the case of green fluorescent protein, 3.2 mg of protein per ml of wheat germ extract can be synthesized using the bi-layer method, and 20 mg can be synthesized using the dialysis method (Harbers 2014). By combining these developed technologies with wheat germ extract, they were able to establish a highly efficient and robust cell-free protein synthesis system for wheat germ for the first time (Figure 1). One of the advantages of the cell-free protein synthesis from wheat germ is the highest success rate in synthesizing functional proteins. In general, ribosomes from eukaryotes, for example, from plants, are better adapted to protein folding during synthesis than ribosomes from prokaryotes, such as those derived from *E. coli*, especially when the target proteins are of eukaryotic origin (Endo 2021). The method for producing wheat germ extract has been published (Madin et al. 2000; Takai et al. 2010), allowing laboratories to prepare germ extracts and establish cell-free translation systems. However, wheat germ extracts prepared using the same process are already commercially available. In addition, wheat germ extracts pretreated with glutathione or metal chelate resins to remove nonspecific binding proteins are also commercially available. Additionally, wheat germ cell-free systems optimized for a variety of applications are commercially available, and their use has the potential to accelerate biochemical studies. These wheat germ cell-free system products are available from CellFree Sciences Co., Ltd. (Yokohama, Japan) (https://www.cfsciences.com).

## Membrane protein production

Membrane proteins play important roles in a variety of biological processes, including signal transduction, substrate transport, energy production, and metabolism, and they account for up to 30% of the genes in the genome (Arabidopsis Genome Initiative 2000; International Human Genome Sequencing Consortium 2001). The production of highly pure and large quantities of membrane proteins is essential for the functional analysis of membrane proteins, but many membrane proteins are difficult to express with existing large-scale protein expression systems using living cells. Recently, cell-free protein synthesis systems, including the wheat germ cell-free system, have gained attention as useful tools for membrane protein production (Sachse et al. 2014; Schwarz et al. 2008; Shinoda et al. 2016). Excessive membrane protein synthesis has significant effects on the physiological functions of host cells (Wagner et al. 2006), but these problems can be overcome in cell-free systems. Additionally, cell-free systems offer the unique advantage of being open format reactions, allowing easy modification of protein synthesis by adding auxiliary components such as lipids and detergents (Fogeron et al. 2021). The simplest method is to add liposomes made from azolectin derived from soybeans (a phospholipid mixture) to the wheat germ extract and synthesize the protein (Nozawa et al. 2007). This method has been shown to be able to synthesize many membrane proteins in a functional state, including plant phosphoenolpyruvate/phosphate translocator, plant aquaporin, human G protein-coupled receptors, and human voltage-gated potassium ion channels (Nemoto et al. 2022; Nishiguchi et al. 2022; Nozawa et al. 2007, 2011; Takeda et al. 2015). Notably, approximately 80% of the 47 recombinant human voltage-gated potassium ion channels tested were found to be functional (Nishiguchi et al. 2022), suggesting that this method is an effective approach for the stable synthesis and functional analysis of membrane proteins. However, although this approach is theoretically very attractive, it must be noted that not all proteins can be incorporated into liposomes, and the function of some membrane proteins is influenced by lipid composition (Levental and Lyman 2023; Nilsson et al. 2016; Stieger et al. 2021).

## Protein array

**Figure 2:**
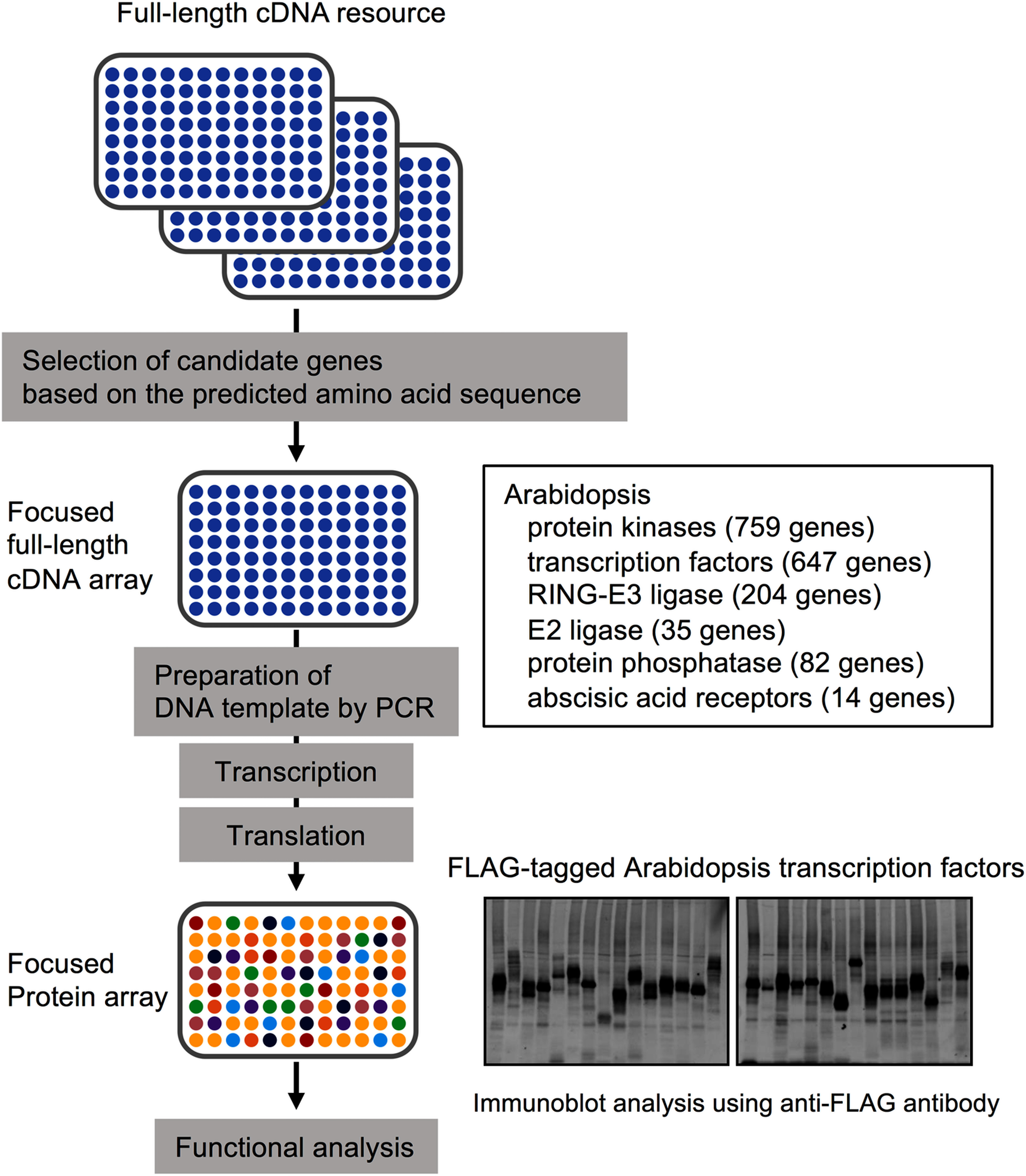
Figure 2. Construction of a protein array using a wheat germ cell-free system. First, candidate genes are selected from cDNA resources such as the RIKEN Arabidopsis full-length cDNA clone (RAFL clone) and a focused full-length cDNA array is created. Next, expression templates are prepared by PCR amplification using the cDNA clones as a template. After the in vitro transcription reaction using expression templates, protein arrays are synthesized using a wheat germ cell-free system. We constructed multiple arrays of RAFL clones that focused on the predicted functions of gene products (e.g. protein kinases and transcription factors).

The high synthetic efficiency inherent in a wheat germ cell-free system is suitable for the synthesis and functional analysis of a wide range of proteins with different biochemical properties. Furthermore, as an in vitro format, the wheat germ cell-free system allows for easy scaling of reactions, automation of experimental procedures, and processing of large numbers of samples, making it a viable option for the development of large-scale protein arrays. Protein arrays are mainly used for comprehensive analysis of biochemical properties and for analyzing signaling networks based on protein–protein interactions. Their biggest advantage is that protein functions can be analyzed in parallel (Sutandy et al. 2013). In a typical protein array, synthesized proteins are immobilized on a solid surface such as a microscope slide, membrane, bead, or microtiter plate (Kingsmore 2006; Sutandy et al. 2013). However, protein immobilization often leads to structural changes and destabilization of the protein, which can result in the loss of its function. Therefore, we circumvented this problem by synthesizing proteins in 96 or 384-well microtiter plates and aliquoting the reaction mixture into small volumes and storing them frozen, or by synthesizing proteins each time (Sawasaki et al. 2002b). By combining wheat germ cell-free systems and cDNA resources, protein arrays covering almost every gene in the human genome have recently been established (Goshima et al. 2008; Morishita et al. 2019). Focused protein arrays have been established in plants, that are selected based on biochemical properties such as protein kinases, protein phosphatases, transcription factors, and others (Figure 2) (Nemoto et al. 2011; Nozawa et al. 2009; Ramadan et al. 2015; Takahashi et al. 2012).

In recent years, highly sensitive quantitative mass spectrometry has been used to estimate the amount of protein present in plants (Heinemann et al. 2021). According to this report, the copy numbers of proteins localized in the cytoplasm range from 114 million molecules for GTP-binding elongation factor Tu family proteins to 2,039 molecules for acetyl-CoA carboxylase 1. Taking into account the detection limit of mass spectrometers, it is estimated that the molecular ratio between the proteins present in the cytoplasm is at least 50,000-fold or more. In contrast, protein arrays synthesized in cell-free wheat germ exhibit differences in protein abundance between arrays that are up to 100-fold, suggesting that the variation is significantly smaller compared to protein ratios in living cells (Goshima et al. 2008). Therefore, by using a detection system with a certain degree of dynamic range, it is possible to cover the entire array for comparison with one detection system. In this way, protein arrays have the advantage of being able to parallelize and analyze the functions of proteins that are present in minute amounts in biological samples or expressed only in a limited number of cells, thereby compensating for the weaknesses of cell-based systems. However, it is essential to consider the quality of the recombinant proteins incorporated into the protein array. As the proteins in the protein array are ectopically expressed recombinant proteins, their structures may not match those of in vivo proteins. In particular, proteins produced in wheat germ cell-free systems often have disulfide bonds and post-translational modifications that are different from those of cell-expressed proteins, so proteins whose post-translational modifications are important for structure and function may behave differently than in vivo (Shields and Blobel 1977; Yamaoka et al. 2022).

## High-throughput screening of substrate proteins and biochemical characterization

Plants have complex mechanisms to adapt to environmental stimuli by inducing physiological and morphological changes in response to environmental changes, and these are primarily caused by qualitative or dynamic changes in a myriad of proteins called intracellular signal transduction systems (Burkart et al. 2022; Nykiel et al. 2022). Post-translational modifications such as phosphorylation and ubiquitination are one of the most important control systems that regulate protein levels, protein localization, and protein activity and are considered front line of the signal transduction system (Miricescu et al. 2018; Sadanandom et al. 2012; Skalak et al. 2021; Zhang et al. 2023). Genome analysis has shown that some plants have more protein kinase and ubiquitin ligase genes than mammals (Buetow and Huang 2016; Hua and Vierstra 2011; Lehti-Shiu and Shiu 2012). However, as the target molecules have not been identified, the molecular mechanisms and signaling networks of their regulatory mechanisms are still largely unknown.

Biochemical approaches such as yeast two hybrid (Y2H) and immunoprecipitation mass spectrometry (IP-MS) are often used to identify target molecules (Brückner et al. 2009; Budayeva and Cristea 2014). Although Y2H does not require special equipment and has a simple experimental process, it often has high false-positive and false-negative rates. IP-MS can comprehensively analyze proteins that directly or indirectly interact with target molecules, but Y2H is often chosen because mass spectrometers are expensive. Furthermore, in many cell-based assays, the level of expression of the target protein within the cell has a major impact on the accuracy of the analysis. Moreover, although recombinant proteins synthesized in a wheat germ cell-free system can be applied to conventional assays such as in vitro kinase assay and pull-down assay, these assays often require cumbersome experimental procedures and have limited throughput (Kanchiswamy et al. 2010; Nemoto et al. 2017; Takahashi et al. 2012).

**Figure 3:**
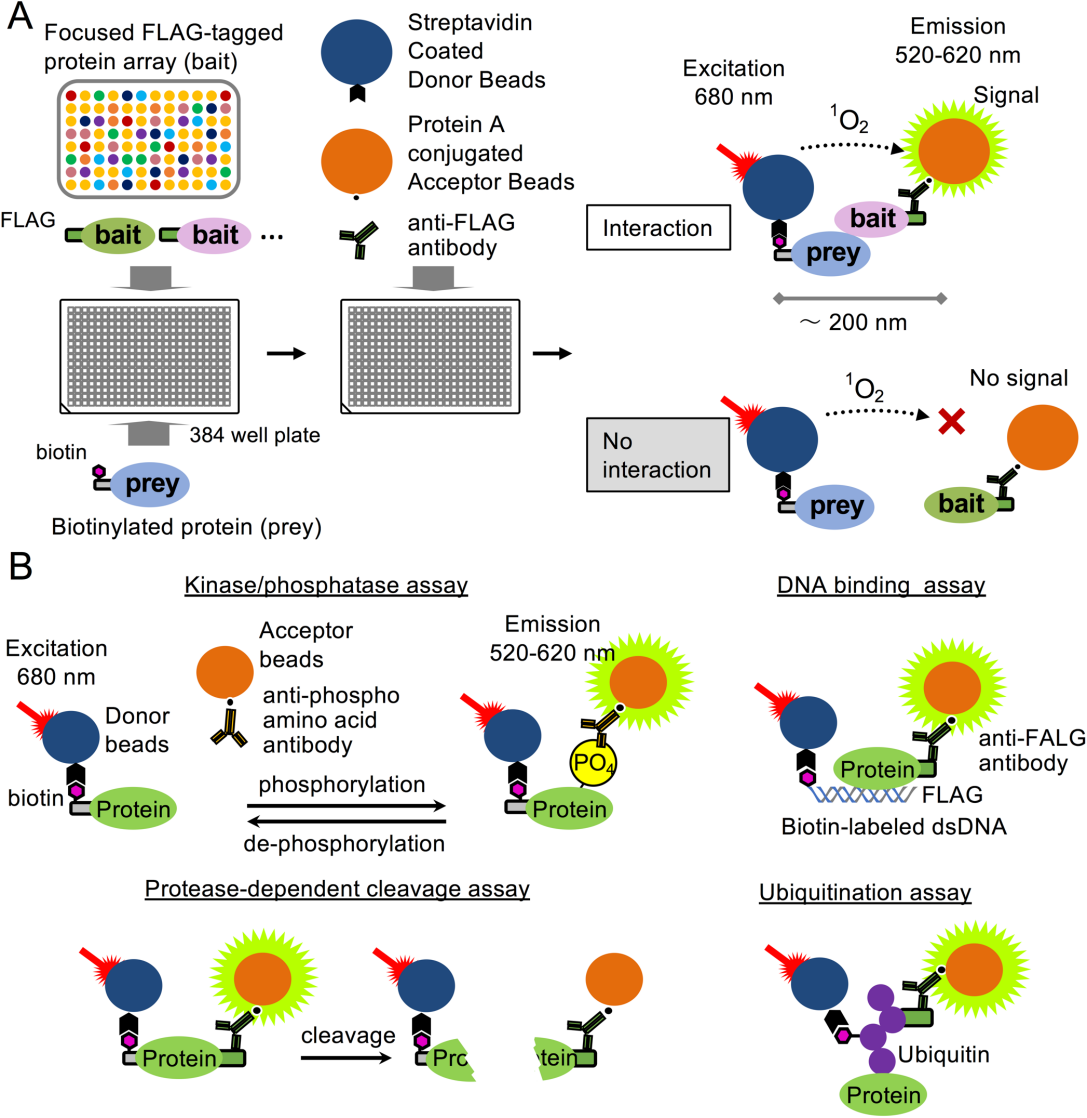
Figure 3. Diverse protein function analysis by combining the wheat germ cell-free system and AlphaScreen. (A) Overview and principles of protein–protein interaction analysis. When a protein–protein interaction occurs between biotinylated protein (prey) and FLAG-tagged target protein (bait), a complex is formed that includes two types of AlphaScreen (top right), streptavidin-coated donor beads and protein A conjugated acceptor beads, contains as well as the interaction signal can be detected by chemical luminescence. In contrast, no interaction signal is detected when there is no interaction between proteins (bottom right). Biotinylated or epitope-tagged proteins are specifically recognized by two types of beads, allowing the use of crude proteins. Previous studies have reported a method to synthesize biotin-labeled proteins using a wheat germ cell-free system (Sawasaki et al. 2008). (B) Design of assay systems for proteins with diverse functions. A schematic diagram for the analysis of protein phosphorylation and dephosphorylation (top left), DNA binding activity (top left), protease-dependent protein cleavage (bottom left), and poly-ubiquitination (bottom left) was presented.

To solve these problems, we have developed a high-throughput target molecule screening method that combines a protein array and the homogeneous assay system AlphaScreen™ technology (PerkinElmer, Inc.) (Figure 3A). AlphaScreen is a chemical luminescence analysis system that relies on the proximity between two types of beads, called “donor” and “acceptor” beads. To summarize the principle of this system, bait and prey proteins are captured by donor and acceptor beads, respectively. When the interaction between bait and prey proteins is at a distance of less than 200 nm, excitation of the donor produces singlet oxygen, which in turn stimulates the acceptor bead to produce a signal (Figure 3A). As the diameter of bovine serum albumin with a molecular weight of approximately 67 kDa is approximately 7 nm (Squire et al. 1968), larger complexes in the range of 200 nm can also be detected. The advantages of this method include low background, high sensitivity, high throughput, wide dynamic range, and a simple experimental procedure that only requires mixing the solutions. In fact, this method has led to the discovery of novel phosphorylated and ubiquitinated substrate proteins involved in various physiological processes such as phytohormone signaling wound response, and proven its effectiveness (Ali et al. 2019; Nemoto et al. 2015, 2017; Takagi et al. 2016; Takaoka et al. 2022). Furthermore, AlphaScreen is capable of not only performing protein–protein interaction analysis but also a variety of functional analyzes tailored to biochemical properties (Figure 3B) (e.g. transcription factor-DNA binding (Nomura et al. 2019), protein kinase activity (Nemoto et al. 2011, 2015), protein phosphatase activity (Nemoto et al. 2015), protease cleavage activity (Tadokoro et al. 2011), polyubiquitination activity (Takahashi et al. 2009)). A wheat germ cell-free system is suitable for protease and ubiquitination assays because hardly any protein degradation activity can be detected in wheat germ extracts (Takahashi et al. 2009). However, it should be noted that some enzymatic activity remains in the wheat germ extract (Nemoto et al. 2017; Ramadan et al. 2015; Takahashi et al. 2009). For example, the wheat germ extracts are contaminated with endogenous unknown protein phosphatases (Kawarasaki et al. 1998), which requires caution when testing with pseudo-substrates such as phospho-peptides. Understanding the various enzyme activities in wheat germ extracts is fundamental to the study of post-translational modifications of proteins; however, in many cases, this problem can be overcome by purifying the synthesized protein.

## Screening of secondary metabolic enzymes

Plants produce useful secondary metabolites that have been used since ancient times as raw materials for chemicals, foods, cosmetics, and pharmaceuticals (Dias et al. 2012). Secondary metabolites are often derived from natural sources or chemically synthesized, but many useful secondary metabolites have complex structures and are difficult to synthesize chemically. Synthetic biological and metabolic engineering approaches may be one way to overcome these problems, but so far, there is limited information about the biosynthetic pathways of secondary metabolites (Afendi et al. 2012; Caspi et al. 2020). In recent years, cell-free translation systems have been used as an efficient method for rapid screening of metabolic enzymes (Jiang et al. 2018; Rasor et al. 2022). In particular, the integration of wheat germ cell-free systems with transcriptomics, comparative genomics, and structural biology approaches could be a valuable approach for screening and biochemical characterization of metabolic enzymes, as well as for enzyme engineering and de novo design (Kido et al. 2020; Kuroiwa et al. 2022; Maeda et al. 2019; Sasaki et al. 2021; Takamura et al. 2021). These open-format systems facilitate easy handling, monitoring, optimization, and sampling and also offer the advantage of simplifying and analyzing complex biosynthetic pathways in vivo. However, many cell-free systems based on extracts from sources such as wheat germ, *E. coli*, and *tobacco* BY-2 contain numerous unknown endogenous enzymes (Buntru et al. 2022; Dudley et al. 2019; Sasaki et al. 2021). Therefore, caution should be exercised when using synthesized proteins without purification. Cell-free systems that store only the components necessary for protein synthesis could be effective in addressing this problem. However, to date, no other cell-free systems have been developed other than the *E. coli*-based PURE system (Shimizu et al. 2001, 2005).

## Chemical screening

Analysis of protein functions using biochemical approaches is important for elucidating the molecular mechanisms of various physiological processes in plants. In particular, the insights gained will provide important information for next-generation molecular breeding, such as genome editing technology. However, protein functional information is also used for the development of novel plant growth regulators.

**Figure 4:**
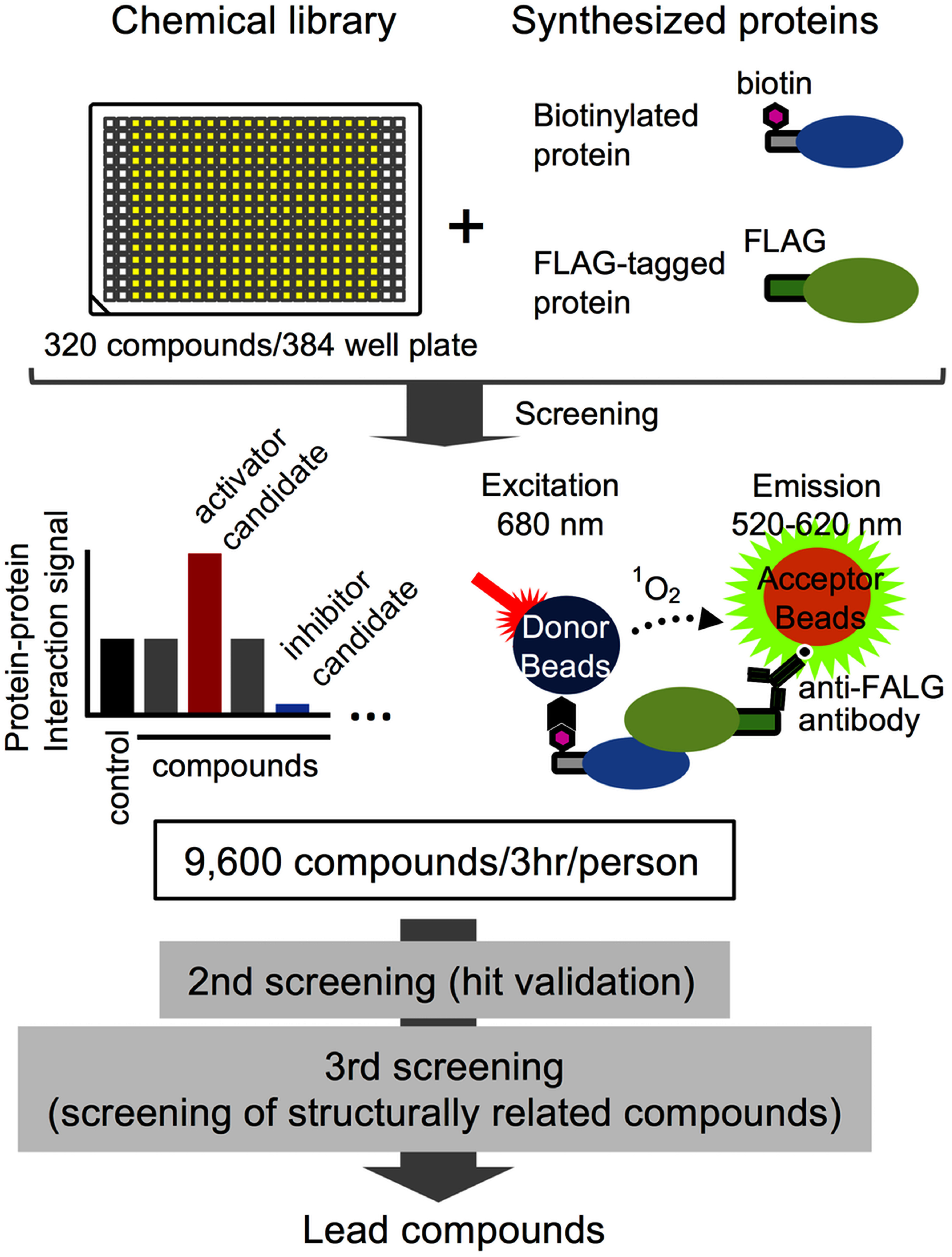
Figure 4. Overview of high-throughput in vitro chemical screening using wheat germ cell-free system and AlphaScreen. Displays an overview of compound screening based on protein–protein interaction analysis using AlphaScreen. Biotinylated or FLAG-tagged proteins synthesized in a wheat germ cell-free system are incubated in the presence of compounds, and interactions between these proteins are analyzed using AlphaScreen. Compounds showing an increase or decrease in protein–protein interaction signal are considered as candidates for activators or inhibitors, respectively. Further secondary and tertiary screening of candidate compounds is conducted to identify functional lead compounds. This system has a high throughput capability, allowing for the analysis of 9,600 compounds within 3 h.

In recent years, target-based chemical screening approaches targeting proteins involved in plant hormone biosynthesis, metabolism, and signaling pathways have attracted attention (Jiang and Asami 2018). Many target-based chemical screening methods utilize the Y2H system to screen compounds that target plant hormone signaling factors such as receptors (Chini et al. 2021; Park et al. 2009; Yoon et al. 2013). However, these approaches have some fundamental limitations. Chemical screening methods using live cells often have problems such as membrane permeability and the toxicity of compounds. In addition, it is difficult to identify the target molecule of the compound in the screening method using plant phenotype as an indicator. In addition, low-throughput is also an important issue in chemical screening. Therefore, an in vitro technique that can search for a compound that directly acts on a target molecule is useful for compound development. To solve these problems, we developed a cell-free screening system for small-molecules that affect protein–protein interactions (Figure 4) (Nemoto et al. 2018). This technology is based on the principle of highly sensitive protein–protein interaction analysis using AlphaScreen, as described above. Chemical screening using AlphaScreen targeting the abscisic acid receptor has been reported in several previous studies, in which receptor proteins synthesized in an *E. coli* system were purified and used for assays (Cao et al. 2013; Melcher et al. 2009, 2010; Ye et al. 2017). In contrast, the results of functional analysis of ABA receptors using a wheat germ cell-free system showed that our system does not require protein purification and is more sensitive compared to assay systems from previous studies (Nemoto et al. 2018). Furthermore, our cell-free based screening system demonstrated high quality and high throughput performance by directly using the translation mixture from the wheat germ cell-free system without protein purification (Figure 4) (Nemoto et al. 2018). In fact, using this method, we discovered novel analogue compounds from a core library of 9,600 chemicals (University of Tokyo Drug Discovery Initiative) that activate abscisic acid receptors in Arabidopsis and gibberellin receptors in grapes, respectively (Nemoto et al. 2018; Nozawa et al. 2023). Nevertheless, by optimizing the assay system, it was shown that it is possible to screen compounds based on indicators such as the interaction between DNA and transcription factors (Nomura et al. 2019). Based on these findings, this method has the potential to become an effective approach for the development of novel plant growth regulators that target key molecules that control physiological processes in plants, such as flowering and senescence, which have significant impacts on agricultural productivity.

## Prospective and future directions

Cell-free systems were developed as a technology to freely synthesize various proteins in vitro. However, the potential of cell-free systems is maximized not only in protein synthesis and functional analysis but also through organic collaborations with various research fields. For example, the combination of cell-free systems and chemical biology can be effective in the development of novel herbicides and plant growth regulators based on biochemical functional analysis. Wheat germ cell-free system can easily achieve fully automated protein production and enable the synthesis and functional analysis of many mutant proteins. Therefore, by integrating the wheat germ cell-free system with bioinformatics and applying synthetic biology techniques, new approaches to optimizing protein function are expected to advance rapidly. This approach may be useful for custom breeding techniques that employ genome editing technology and for the production of useful secondary metabolites at an industrial level using bacterial cells. Plant pathogens cause significant economic damage and are increasing worldwide. To prevent the spread of the disease, it should ideally be diagnosed on the field using a simple and quick method such as an immunochromatographic assay. High-quality antigen molecules are required for antibody production. However, some proteins, such as viruses, are difficult to synthesize in living cell systems. It has been shown that the wheat germ cell-free system can efficiently synthesize viral proteins and use them as antigens to produce highly sensitive and specific antibodies (Goto et al. 2020; Matsunaga et al. 2014; Yamaoka et al. 2016). Therefore, antibody production that utilizes wheat germ cell-free systems can potentially accelerate the development of virus diagnostic kits. As the wheat germ cell-free system has proven to be one of the most effective cell-free eukaryotic systems, we hope that the wheat germ cell-free system will contribute not only to these developments but also to plant biotechnology more broadly.

## Abbreviations

*E. coli*: *Escherichia coli*
IP-MS: immunoprecipitation mass spectrometry
Y2H: yeast two hybrid
